# Impact of fluoroquinolones and aminoglycosides on *P. aeruginosa* virulence factor production and cytotoxicity

**DOI:** 10.1042/BCJ20220527

**Published:** 2022-12-22

**Authors:** Daniel M. Foulkes, Keri McLean, Marta Sloniecka, Sophie Rustidge, Dominic P. Byrne, Atikah S. Haneef, Craig Winstanley, Neil Berry, David G. Fernig, Stephen B. Kaye

**Affiliations:** 1 Department of Eye and Vision Science, Institute of Ageing and Chronic Disease, University of Liverpool, Liverpool, U.K.; 2 Department of Biochemistry, Institute of Integrative Biology, University of Liverpool, Liverpool, U.K.; 3 Department of Clinical Infection, Institute of Infection and Global Health, University of Liverpool, Liverpool, U.K.; 4 Department of Chemistry, University of Liverpool, Liverpool, U.K.

**Keywords:** aminoglycosides, ExoS, ExoU, fluoroquinolones, *Pseudomonas aeruginosa*, type III secretion system

## Abstract

The opportunistic pathogen *Pseudomonas aeruginosa* is one of leading causes of disability and mortality worldwide and the world health organisation has listed it with the highest priority for the need of new antimicrobial therapies. *P. aeruginosa* strains responsible for the poorest clinical outcomes express either ExoS or ExoU, which are injected into target host cells via the type III secretion system (T3SS). ExoS is a bifunctional cytotoxin that promotes intracellular survival of invasive *P. aeruginosa* by preventing targeting of the bacteria to acidified intracellular compartments. ExoU is a phospholipase which causes destruction of host cell plasma membranes, leading to acute tissue damage and bacterial dissemination. Fluoroquinolones are usually employed as a first line of therapy as they have been shown to be more active against *P. aeruginosa in vitro*than other antimicrobial classes*.* Their overuse over the past decade, however, has resulted in the emergence of antibiotic resistance. In certain clinical situations, aminoglycosides have been shown to be more effective then fluoroquinolones, despite their reduced potency towards *P. aeruginosa in vitro*. In this study, we evaluated the effects of fluoroquinolones (moxifloxacin and ciprofloxacin) and aminoglycosides (tobramycin and gentamycin) on T3SS expression and toxicity, in corneal epithelial cell infection models. We discovered that tobramycin disrupted T3SS expression and reduced both ExoS and ExoU mediated cytotoxicity, protecting infected HCE-t cells at concentrations below the minimal inhibitory concentration (MIC)_._ The fluoroquinolones moxifloxacin and ciprofloxacin, however, up-regulated the T3SS and did not inhibit and may have increased the cytotoxic effects of ExoS and ExoU.

## Introduction

*Pseudomonas aeruginosa* is Gram-negative bacterium that colonises a diverse range of environmental niches. *P*. *aeruginosa* is also a major opportunistic pathogen and common cause of nosocomial infection, associated with a wide range of diseases, including pneumonia and microbial keratitis [1–3]. It is a leading cause of intensive care unit-acquired pneumonia (ICUAP) [4], and is the second most frequent colonising bacteria in patients with COVID-19 [5,6]. It is also the primary causative agent of bacterial keratitis, which is recognised as the second largest cause of legal blindness worldwide [7]. As a pathogen of current major concern, the world health organisation (WHO) has listed carbapenem-resistant *P. aeruginosa* (CRPA) with the highest priority for the development of new antimicrobial therapies [8].

Pathogenic *P. aeruginosa* strains use the type III secretion system (T3SS), to inject exotoxins directly into the cytoplasm of compromised host epithelia [9]. The T3SS has been identified as a principal virulence determinant for poor clinical outcomes in pneumonia, sepsis, keratitis, and otitis externa [2–4,10,11]. T3SS expressing *P. aeruginosa* clinical isolates can be further categorised as either exotoxin S (ExoS) or exotoxin U (ExoU) producing. In a study of hospitalised patients with *P. aeruginosa* bacteraemia, 97.5% of bloodstream isolates were positive for *exoU* or *exoS* genes, with isolates containing *exoU* being significantly more resistant to antibiotic treatment [12]. ExoS ADP-ribosyl transferase (ADPRT) activity catalyses ADP-ribosylation of distinct human target proteins, including Rac, Rho and Ras, inducing cytoskeletal disorder, breakdown of cell junctions, inhibition of autophagy and eventual cell death, leading to persistent infections [13]. ExoS ADPRT activity prevents endosome maturation and intracellular membrane trafficking, allowing *P. aeruginosa* to exploit an intracellular replicative niche [14,15]. ExoU is a ubiquitin activated phospholipase that localises to the inner leaflet of host cell plasma membranes (via phosphatidylinositol 4,5-bisphosphate (PIP_2_) dependent targeting) where it induces cytolysis by cleaving phospholipids[16]. ExoU catalytic activity is directed towards phospholipids at the sn-2 position, and results in arachidonic acid release which induces pathways that result in NF-κB activation and MAPK signalling [17–19]. This leads to up-regulation of IL-8 and keratinocyte chemoattractant (KC), and increased infiltration of neutrophils that exacerbate tissue damage via acute localised inflammation [17,18].

T3SS expression and production of ExoS and ExoU is tightly controlled at the transcriptional level in response to environmental cues, including contact with host cells and low levels of extracellular calcium ions [19,20]. Expression is controlled principally by the interactions of four transcription factors: ExsA, ExsC, ExsD, and ExsE, with the AraC family transcription factor, ExsA serving as the primary activator of *P. aeruginosa* T3SS gene expression [21]. ExsA DNA biding induces expression of several proteins that form the T3SS macromolecular complex, spanning the inner bacterial membrane, the periplasmic space, the peptidoglycan layer, the outer bacterial membrane, the extracellular space, and the host cell membrane [9]. The needle like structure is assembled by helical polymerisation of PscF proteins [22]. PcrV is an essential translocator protein for exotoxin secretion which forms the T3SS needle tip [23].

T3SS expression in *P. aeruginosa* is associated with acute toxicity, and delay of- or failure to initiate adequate antimicrobial therapy is linked to increased mortality [10]. Fluoroquinolones, such as moxifloxacin and ciprofloxacin, disrupt bacterial DNA replication by inhibiting DNA topoisomerases and DNA-gyrases, and are normally the primary line of treatment for *P. aeruginosa* infections [24]. They demonstrate high potency against most clinical isolate strains of *P. aeruginosa in vitro*, however, their use has led to the emergence of *P. aeruginosa* strains with resistant phenotypes, predominantly via efflux dependent mechanisms [25]. Aminoglycosides are another group of antimicrobial agent used in the treatment of *P. aeruginosa* infections, which function by binding to the A-site (aminoacyl) of 16S rRNA, a component of the bacterial ribosomal 30S subunit, to disrupt protein synthesis [26]. In comparison with fluoroquinolones, aminoglycosides (such as tobramycin, amikacin, and gentamycin) are generally considered less potent compounds towards *P. aeruginosa* when assayed *in vitro*; however, they can demonstrate improved utility against *P. aeruginosa* infections in certain clinical settings. For example, inhalation of aerosol formulations of aminoglycosides, especially tobramycin, have proven efficacious in the treatment and prevention of bronchiectasis [27]. Despite being a major determinant in disease progression and clinical outcome, the effects these antimicrobials have on T3SS expression (if any) is currently unknown. In this study we explore the mechanisms through which such antimicrobials might influence *P. aeruginosa* T3SS-dependent toxicity, as a potential determinant for informing choice of treatment, particularly in situations where the MIC may not be achieved.

## Results

### Analysis of fluoroquinolones and aminoglycosides on *P. aeruginosa* growth

**Figure 1.**
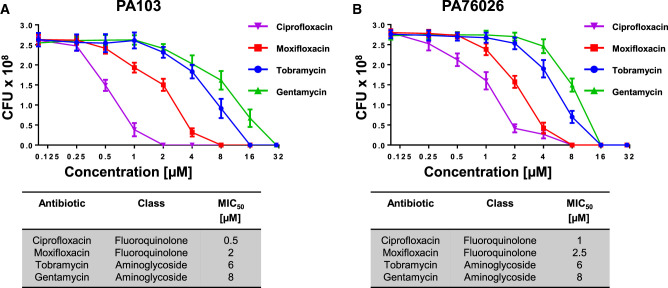
Antibiotic minimal inhibitory concentrations for ExoU expressing PA103 and ExoS expressing PA76026 strains of *P. aeruginosa*. The antibiotic at 50% minimal inhibitory concentration (MIC_50_) for ExoU expressing PA103 (**A**) and ExoS expressing PA76026 (**B**) strains of *P.aeruginosa* were determined by measuring absorbance reading at OD_600nm_ to assess bacterial growth, after cultures were incubated with varying concentrations of specified antibiotic in 96-well plates. The antibiotic type and their MIC_50_ for growth of PA103 and PA76026 are displayed.

Our aim was to analyse the effects of antimicrobials on T3SS virulence factor expression below their respective minimal inhibitory concentrations (MICs). For this purpose, we first established the MIC_50_ (concentration required for 50% bacterial growth inhibition) for two fluoroquinolones (moxifloxacin and ciprofloxacin) and two aminoglycosides (tobramycin and gentamycin) on the growth of the *P. aeruginosa* strains PA103 and PA76026. PA103 expresses ExoU whereas PA76026 expresses ExoS. This revealed that both *P. aeruginosa* strains were more susceptible to inhibition by the fluoroquinolones than the aminoglycosides after 16 h of growth (Figure 1A,B). MIC_50_ for ciprofloxacin, moxifloxacin, tobramycin and gentamycin were determined to be 0.5, 2, 6, and 8 µM for PA103 and 1, 2.5, 6 and 8 µM for PA76026, respectively.

### Effects of antimicrobials on PcrV expression in *P. aeruginosa*

Western blotting was used to detect changes in expression of the essential T3SS needle tip component, PcrV [9], in PA103 and PA76026 after 16 h incubation in the presence of antimicrobials at their respective MIC_50_, and using an antibody with specificity towards PrcV (Figure 2A). Although less bactericidal than ciprofloxacin and moxifloxacin (Figure 1), the aminoglycoside tobramycin (6 µM) caused a sharp reduction in total PcrV for both PA103 (∼74.0% reduction) and PA76026 (∼50.5% reduction) (*P* = 0.001 and 0.003). Gentamycin, also an aminoglycoside, did not detectably alter PcrV expression for either cell line. The fluoroquinolone moxifloxacin, however, caused a statistically significant increase in the relative abundance of PcrV in PA103 (81.8% increase, *P* = 0.004), whereas ciprofloxacin, caused a similar increase in PcrV expression in PA76026 (57.0%, *P* = 0.003) (Figure 2A).

**Figure 2.**
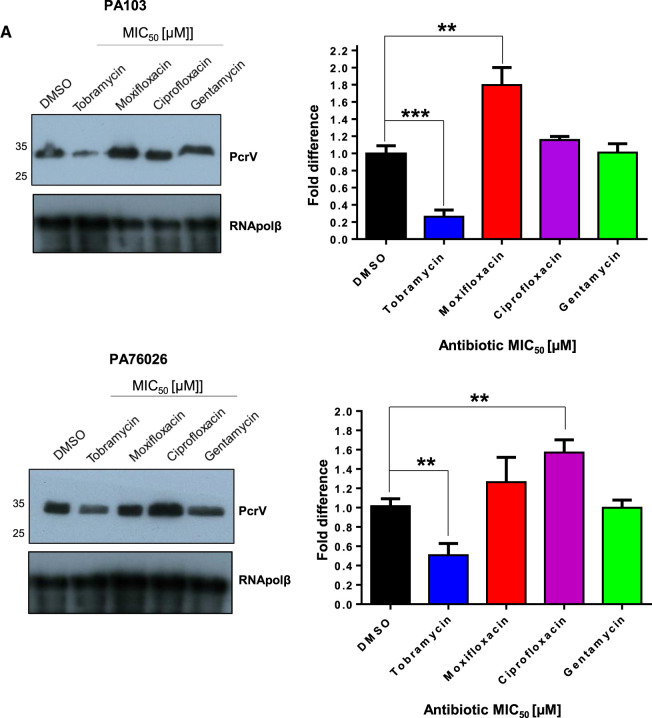
Effects of antibiotics on PcrV expression in *P. aeruginosa*. Part 1 of 2 (**A**) Expression of PcrV in PA103 and PA76026 after 16 h incubation with indicated antibiotic at the MIC50, determined by western blotting. Relative band intensities were calculated using imageJ software from three independent experiments, with RNApolβ serving as the loading control. T-tests were used to determine statistically significant difference in relative PcrV expression levels.

**Figure 2.**
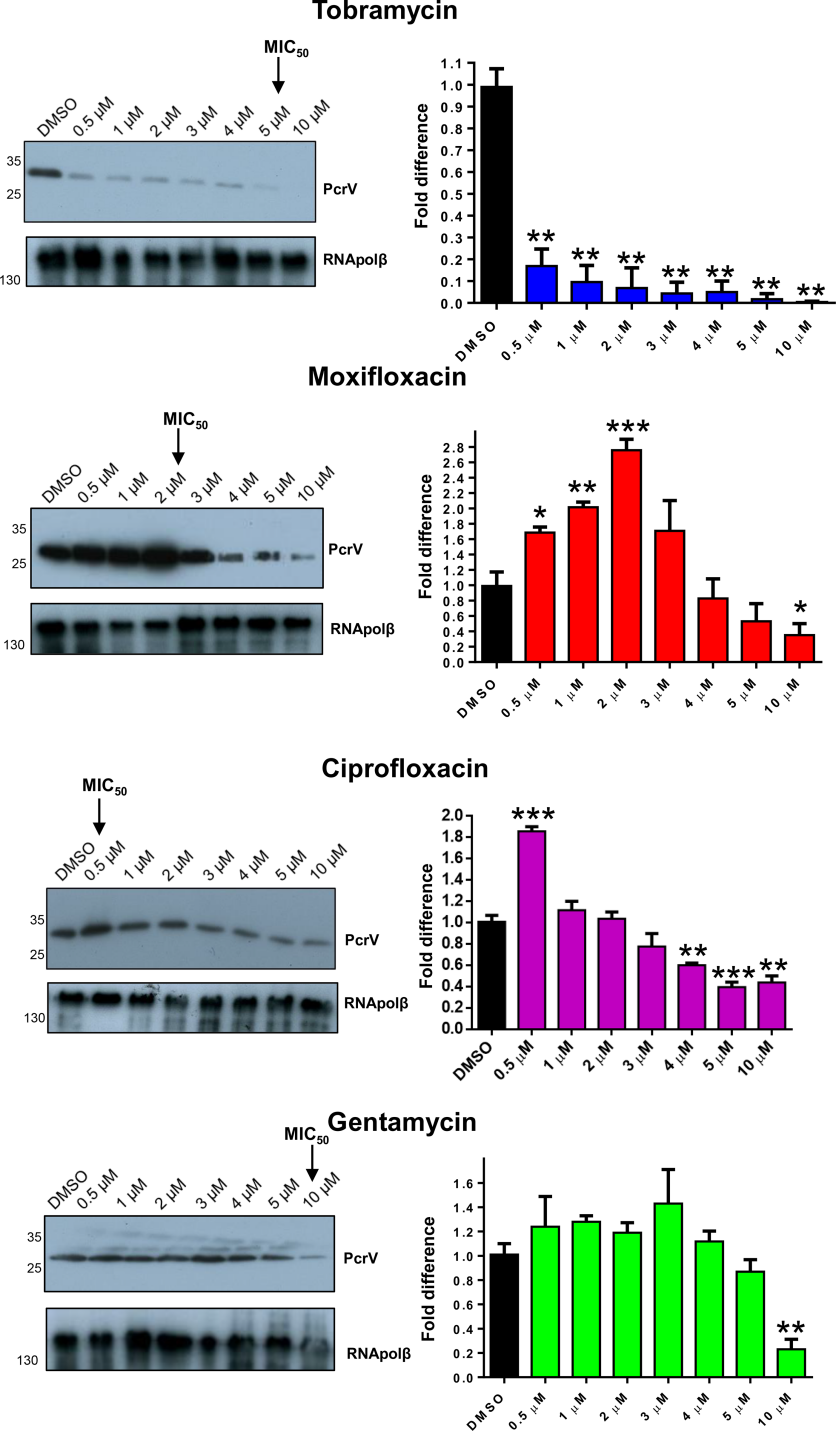
Effects of antibiotics on PcrV expression in *P. aeruginosa*. Part 2 of 2 (**B**) Antibiotic dose response analysis on PcrV expression in PA103 was determined by western blotting. Relative band intensities were determined from three independent experiments, normalised to RNApolβ. T-tests were performed to determine statistically significant changes in PcrV production; *P < 0.05; **P < 0.01; ***P < 0.001.

To evaluate antimicrobial-dependent changes in PcrV expression in more detail, PA103 was exposed to antimicrobials at varying concentrations prior to western blotting (Figure 2B). Even at 0.5 µM (4.2% of the MIC), tobramycin caused a noticeable reduction in detectable PcrV (Figure 2B). In contrast, PcrV abundance increased at moxifloxacin concentrations between 0.5 and 3 µM, and returned to basal levels at concentrations above the MIC_50_ (>2 µM). Ciprofloxacin, which is a more potent antimicrobial than moxifloxacin (MIC_50_ of 0.5 µM compared with 2 µM, respectively), increased PcrV expression at 0.5 µM, but depleted PcrV at concentrations >4 µM. The aminoglycoside gentamycin only induced significant loss of PcrV expression at concentrations above 10 µM (Figure 2B).

### Analysis of T3SS-related gene transcription in *P. aeruginosa* in response to antimicrobial exposure

**Figure 3.**
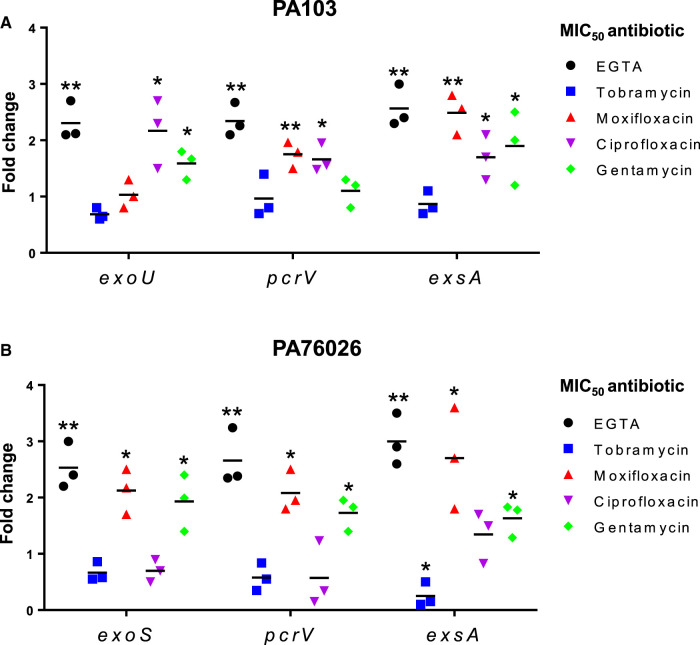
Impact of antibiotics on T3SS gene expression in PA103 and PA76026. PA103 (**A**) and PA76026 (**B**) were incubated for 16 h in the presence of indicated antibiotic (at the MIC_50_) prior to RT-qPCR analysis to detect relative mRNA levels of T3SS associated genes. Incubation with 2 mM of EGTA served as the positive control for T3SS induction. Individual fold change values and means (-) from three independent experiments were plotted; *P < 0.05; **P < 0.01.

To further investigate how antimicrobials impact expression of the *P. aeruginosa* T3SS complex and associated cytotoxins at the transcriptional level, we used RT-qPCR (Real-Time Quantitative Reverse Transcription PCR) to detect changes in mRNA levels for *exoU* (for PA103), *exoS* (for PA76026), *pcrV* and the key T3SS activating transcription factor, *exsA*. EGTA, which has previously been shown to increase *exsA* transcription in *P. aeruginosa*, was used as a positive control to up-regulate T3SS expression [14,28]. After incubation with 2 mM EGTA for 16 h, there was a predictable increase in *exoU* (PA103), *exoS* (PA76026), *pcrV* and *exsA* mRNA in both *P. aeruginosa* isolates (Figure 3A,B, black). With tobramycin, we observed a statistically insignificant 0.7-fold decrease in *exoU* mRNA in PA103, relative to DMSO treated controls (Figure 3A, blue). *pcrV* and *exsA* mRNA levels were also relatively unaffected. With moxifloxacin, *exoU* mRNA levels did not significantly change, however, we did observe 1.8 and 2.5-fold increases for *pcrV* and *exsA* (Figure 3A, red) which is consistent with the changes we previously observed at the protein level in moxifloxacin treated PA103 cells (Figure 2). In the presence of ciprofloxacin, *exoU* transcription increased 2.2-fold, whereas *pcrV* and *exsA* mRNA both modestly increased 1.7-fold (Figure 3A, purple). For gentamycin treated PA103 there were no observable changes in *pcrV* mRNA, although both *exoU* and *exsA* transcription were increased 1.6 and 1.9-fold (Figure 3A, green).

For PA76026, tobramycin had a more pronounced effect decreasing mRNA levels of *exsA* by 0.3-fold (Figure 3B, blue). Conversely, in the presence of moxifloxacin we observed a 2.1-fold up-regulation of both *exoS* and *pcrV*, whilst *exsA* mRNA levels were increased 2.7-fold, similar to that observed for EGTA treatment (Figure 3B, red). For ciprofloxacin treated PA76026 (Figure 3B, purple) there were no statistically significant changes in neither *exoS*, *pcrV* or *exsA* transcripts. In gentamycin treated PA76026 there were consistent modest increases in *exoS*, *pcrV* and *exsA* mRNA levels (1.9, 1.7 and1.6-fold).

### Effect of antibiotics on secreted ExoU and ExoS activity

**Figure 4.**
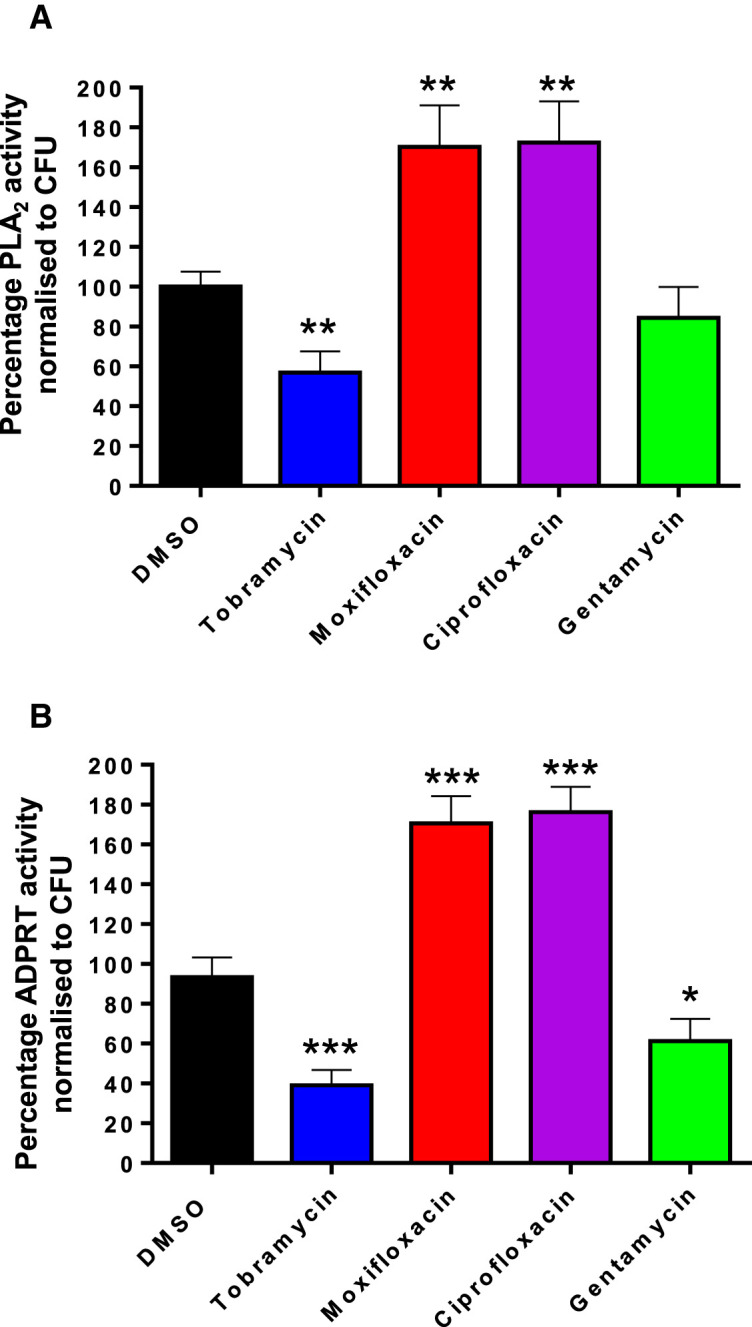
Tobramycin reduces secretion of T3SS proteins ExoU and ExoS. PA103 (**A**) and PA76026 (**B**) were incubated for 16 h in the presence of indicated antibiotic at their respective MIC50. The cleared bacterial culture medium was then assayed for either ExoU activity (**A**), employing a phospholipase assay, or ExoS activity (**B**), employing an ADPRT assay. Phospholipase endpoint assays were ran for 16 h and ADPRT endpoint assays were ran for 4 h. The percentage activity was normalised to bacterial CFU count (Supplementary Figure S2), with reference to DMSO (100% activity) treated controls. With reference to DMSO and normalised to bacterial CFU count. Bars represent means from three independent experiments. T-tests were employed to determine statistically significant changes relative to DMSO treated *P. aeruginosa*; *P < 0.05; **P < 0.01; ***P < 0.001.

Since tobramycin reduced T3SS expression, as judged by a diminished PcrV protein signal (Figure 2) and reduced *exsA* transcript levels in PA76026 (Figure 3B), we next probed for accompanying modulation in ExoU (PA103) and ExoS (PA76026) secretion (Figure 4). PA103 (Figure 4A) and PA76026 (Figure 4B) were incubated with either tobramycin, moxifloxacin, ciprofloxacin and gentamycin (at the respective MIC_50_) for 16 h after which point, the cleared culture medium (by centrifugation at 5000***g*** for 10 min) was analysed using either a phospholipase assay or ADPRT assay. Enzymatic activity was detected with reference to DMSO controls and given that the application of antimicrobials reduced bacterial growth and that the number of bacterial CFUs may not predict the amount of exotoxin present, ExoU and ExoS activity was normalised to the quantity of CFUs detected (Supplementary Figure S1).

In the presence of tobramycin there was a 43.2% decrease in ExoU phospholipase activity detected in the culture medium of PA103 treated cells (Figure 4A). Conversely, both moxifloxacin and ciprofloxacin caused a sharp increases (70.2% and 72.4%) in detectable ExoU activity (Figure 4A). Treatment of PA103 with gentamycin, however, did not cause a statistically significant change in observable secreted ExoU activity (Figure 4A).

Employing recombinant human kRas as a substrate and 14-3-3 ἠ as the ExoS activating co-factor, the ADPRT catalytic activity of secreted ExoS, from PA76026, was assessed 16 h after antibiotic exposure (Figure 4B). With the total percentage activity referenced to untreated DMSO controls (0.01% v/v) and normalised to detected CFU (Supplementary Figure S1B), tobramycin elicited the sharpest reduction in observable ExoS ADPRT activity; 61.0% (Figure 4B, blue). Gentamycin caused a noticeable decrease (38.7%) in secreted ExoS ADPRT activity (Figure 4B, green). Similar to our observation with secreted ExoU activity, moxifloxacin and ciprofloxacin caused stark increases in observable ExoS ADPRT activity (70.6% and 76.2%). Importantly, none of these antimicrobials inhibited the enzymatic activity of recombinant His-tagged ExoU (Supplementary Figure S2A) or His-tagged ExoS (Supplementary Figure S2B) expressed in and purified from *E. coli*, indicating that tobramycin prevented ExoU and ExoS production and/or secretion rather than having a direct inhibitory effect on catalytic activity.

**Figure 5.**
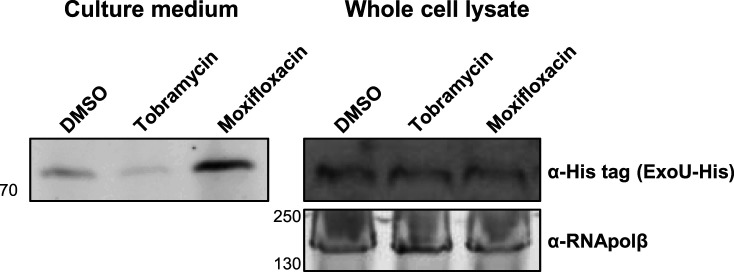
Tobramycin reduces secretion of His-tagged ExoU in PA103. PA103 transformed with a pUCP20T plasmid encoding ExoU-His was incubated with DMSO (0.1% v/v), tobramycin and moxifloxacin, at MIC_50_, for 16 h. The cleared culture medium was then analysed by western blotting, employing an anti-6xhistidine primary antibody, in order to detect secreted C-terminal His-tagged ExoU. Whole cell lysates were also analysed to detect relative levels of intracellular expressed His-tagged ExoU. Total RNApolβ was detected to serve as a loading control.

As (to our knowledge) there are currently no commercially available ExoU antibodies, we transformed PA103 with a pUCP20T plasmid encoding ExoU modified with a C-terminal 6x histidine tag, to serve as an artificial antigen for immunogenic detection. We quantified secreted ExoU-His in the culture medium by western blotting (Figure 5). When PA103:pUCPT20-ExoU-His was incubated overnight with 0.01% (v/v) DMSO, ExoU was readily detected in the medium (Figure 5, left) but not in the presence of tobramycin. In accordance with our previous observation that moxifloxacin causes an increase in secreted ExoU catalytic activity, we observed higher detectable quantities of His-tagged ExoU in the culture medium of moxifloxacin treated PA103 (Figure 5, left). We also detected the total quantity of intracellular ExoU-His in PA103:pUCP20T-ExoU-his whole cell lysates (Figure 5, right) Interestingly, the level of cellular ExoU-His expressed from the pUCP20T plasmid in transformed PA103, was not impacted by the presence of tobramycin (Figure 5, right), supporting an effect of tobramycin on secretion of this exotoxin.

### Effects of antimicrobials on PA103 cytotoxicity in a wound healing infection model

**Figure 6.**
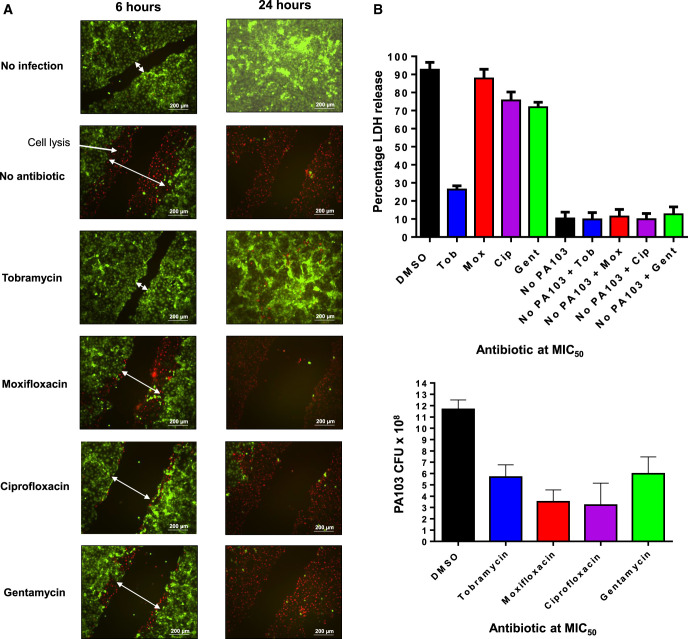
Tobramycin reduces ExoU mediated cytotoxicity in a HCE-t scratch and infection assay. (**A**) Live/Dead fluorescence microscopy analysis of scratched HCE-t cells 6 h (left) and 24 h (right) post infection with PA103 in the presence of indicated antibiotic at the MIC_50_. (**B**) Percentage LDH release from infected HCE-t cells in the presence of antibiotics 24 h post infection. (**C**) Number of PA103 CFUs per ml detected the cell culture medium of HCE-t cells 24 h after infection, with antibiotic present at the MIC50.

In a previous study, we developed a corneal epithelial HCE-t cell scratch and infection assay to evaluate inhibitors of ExoU as an *in vitro* model of disease [29]. Infection and ExoU cytotoxicity is established along the border of the scratch, preventing healing and leading to a widening of the wound, which can be observed by fluorescence microscopy, while ExoU cytotoxicity can simultaneously be indirectly estimated by LDH assays. We set out to determine how antimicrobials might influence acute ExoU-driven cytotoxicity after infection of HCE-t cells with PA103, using LIVE/DEAD fluorescence microscopy analysis to observe HCE-t cell viability and wound healing (Figure 6A) in addition to quantifying cytotoxicity using an LDH assay (Figure 6B).

When scratched HCE-t cells were incubated for 6 and 24 h without PA103 (DMSO 0.01% v/v), we observed wound healing (Figure 6A, top) and background (no apparent toxicity) levels of LDH release (Figure 6B, no PA103). Reciprocally, when PA103 was present, significant toxicity could be detected after 6 h, with almost all of the cells succumbing to infection after 24 h (Figure 6A, no antibiotic). Tobramycin (at MIC_50_) was able to mitigate cytotoxicity and promote total wound closure after 24 h (Figure 6A). This was also reflected by a reduction in LDH release (26.3%) compared with DMSO (Figure 6B). Moxifloxacin, ciprofloxacin and gentamycin partially reduced cytotoxicity during 6 h of infection, which manifest as reduced cell lysis at the scratch border and decreased wound size (Figure 6A). They were, however, ineffective over 24 h (Figures 6A and 5B). To prove that the reduction in observed toxicity for tobramycin treated cells was not an effect of reduced bacterial growth, we detected the number of viable PA103 CFUs in the HCE-t cell culture medium after 24 hours of infection (Figure 6C). Despite the detection of bacterial expansion in all antibiotic treatment conditions, only tobramycin afforded protection from ExoU mediated cytotoxicity.

### Effect of antimicrobials on ExoS cytotoxicity after PA76026 infection

**Figure 7.**
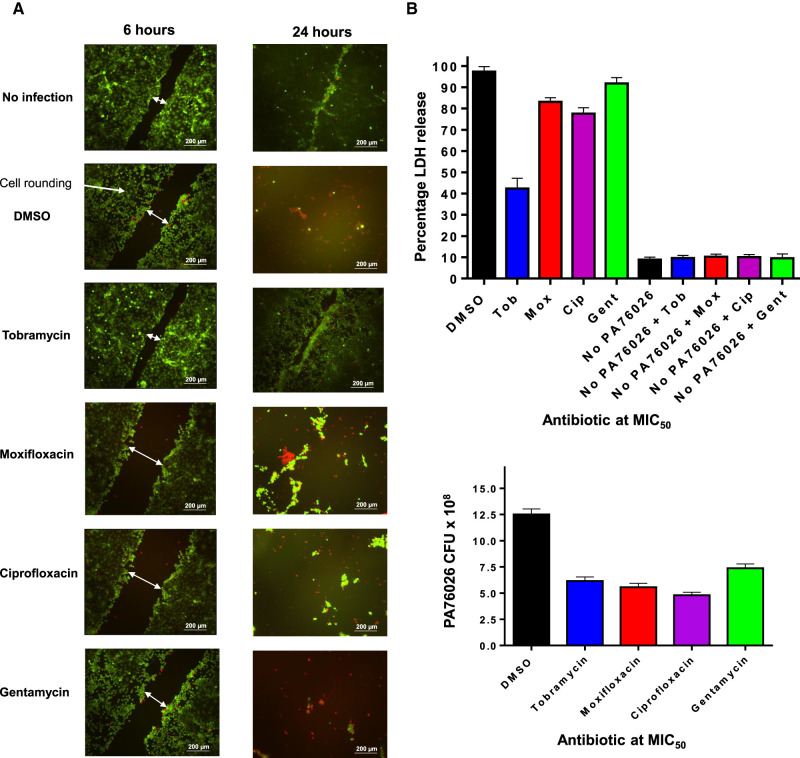
Tobramycin reduces ExoS mediated cytotoxicity in PA76026 during HCE-t cell infection. (**A**) Live/Dead fluorescence microscopy analysis of scratched HCE-t cells at 6 h (left) and 24 h (right) post infection with PA76026 in the presence of indicated antibiotic at the MIC_50_. (**B**) Percentage LDH release from infected HCE-t cells in the presence of antibiotics 24 h post infection. (**C**) Number of PA76026 CFUs per ml detected the cell culture medium of HCE-t cells 24 h after infection, with antibiotic present at the MIC_50_.

We next sought to determine whether the panel of antimicrobials could prevent T3SS mediated cytotoxicity from the ExoS expressing strain of *P. aeruginosa*, PA76026. In our scratch and infection assay, ExoS activity caused cell rounding along the site of the initial scratch from 3 h and more extensively after 6 h (Supplementary Figure S3). Cell rounding in this manner due to ExoS activity has been reported previously [14,30,31]. In the absence of an antimicrobial, cell death occurred after 24 h due to bacterial expansion, which overwhelmed the culture medium and therefore may not be attributed to ExoS action alone (Supplementary Figure S3). The bacterial load was controlled by antimicrobials at their respective MIC_50_, which enabled analysis of ExoS-mediated toxicity in scratched HCE-t cells over 24 h (Figure 7). As before we adopted a combinatorial approach, using LIVE/DEAD fluorescence microscopy to observe cell viability and morphological changes (Figure 7A) and LDH release assay to determine cytotoxicity after 24 h of infection (Figure 7B).

Tobramycin abolished PA76026 mediated cytotoxicity, resulting in no observable cell rounding at 6 h and advanced wound healing after 24 h (Figure 7A). This was accompanied by a 68% reduction in LDH release (Figure 7B). Conversely, moxifloxacin, ciprofloxacin and gentamycin did not facilitate wound healing and we observed numerous rounded cells along the border of the scratch (Figure 7A). Treatment with these antibiotics elicited only slight reductions in LDH release (Figure 7B). Importantly, none of the antimicrobial compounds exhibited cytotoxicity as judged by LDH assay (Figures 6B and 7B). Finally, we detected the number of viable PA76026 CFUs across antimicrobial treatments, 24 h after infection of scratched HCE-t cells (Figure 7C). Bacterial growth was demonstrated to be similar for each antibiotic tested, however, only tobramycin was able to inhibit the effects of ExoS induced cytotoxicity in our scratch and infection assay.

## Discussion

ExoS and ExoU expressing strains of *P. aeruginosa* are related to poorest prognosis in pneumonia and contact lens associated keratitis [2,10,32]. The current treatment for *P. aeruginosa* keratitis is the prescription of multiple antibiotics, which must be introduced rapidly following the onset of symptoms to minimise corneal damage [33]. This approach often results in corneal toxicity and selection for antibiotic-resistance [34], leading to failure of treatment. Therefore, a better understanding of the effects of antimicrobials on *P. aeruginosa* virulence will be critical for developing improved therapeutic strategies.

The aminoglycoside, tobramycin, at concentrations at and below the calculated MIC_50_ caused a statistically significant reduction in the T3SS secretion apparatus protein, PcrV (Figure 2A,B) in both ExoU expressing PA103 and ExoS expressing PA76026 cells, which correlated with diminished ExoU and ExoS secretion (Figure 4). After tobramycin treatment, *exsA* mRNA was significantly reduced in PA76026 (Figure 3B). Reduced expression of the ExsA transcription factor would likely lead to disrupted T3SS assembly which consequentially might explain the depletion of secreted ExoS activity we observed under the same treatment conditions (Figure 4B). This, however, was not the case in tobramycin treated PA103, whereby *exsA* mRNA was unaffected (Figure 3A). Nonetheless, tobramycin-induced loss of PcrV protein (Figure 2), which is required for T3SS effector translocation, could explain the observed reduction in secreted ExoU activity. The other aminoglycosides investigated in this study, gentamycin also reduced PcrV expression, but only at concentrations above the MIC_50_ (Figure 2B), which might suggest either reduced penetration or potency of gentamycin compared with tobramycin.

Aminoglycosides inhibit bacterial protein synthesis [26], and we hypothesised that this was the dominant mode of action to explain the reduced expression of PcrV, ExoS and ExoU that we observed for the two clinical strains of *P. aeruginosa* used in this study. However, we observed that intracellular levels of histidine tagged ExoU expressed from a pUCP20T plasmid by PA103 was unaffected by tobramycin exposure (Figure 5). This would suggest that the observed decrease in virulence factor in the medium after tobramycin treatment resulted from an effect on the secretion system rather than a global inhibition of protein synthesis. Independent studies demonstrate that aminoglycosides can increase biofilm formation and up-regulate quorum sensing in *P. aeruginosa* [35,36]. As RhlR mediated quorum sensing has previously been demonstrated to negatively regulate the T3SS in PA01 [37], a speculative mechanism by which tobramycin reduces T3SS expression could be through influence on RhlR-C4HSL signalling.

Fluoroquinolones, such as moxifloxacin, function by inhibiting bacterial DNA replication by targeting DNA topoisomerase and DNA-gyrase [24]. Here, we observed that moxifloxacin (at sub-lethal concentrations) increased total *exsA* and *pcrV* mRNA in both isolates, and *exoS* in PA76026 cells (but not *exoU* in PA103), which collectively suggested a general up-regulation of T3SS expression that is comparable to the established T3SS inducing agent, EGTA. This also correlated with a concentration dependent increase in PcrV protein for PA103 challenged with moxifloxacin below the MIC_50_. We also observed that moxifloxacin and ciprofloxacin increased ExoU and ExoS secretion (Figure 4). This highlights the concerning possibility that targeting *P. aeruginosa* with fluoroquinolones, particularly at sub-lethal concentrations, might enhance T3SS expression. Previous studies have also demonstrated that sub-inhibitory concentrations of antibiotics can produce specific changes in the behaviour of *P. aeruginosa*. Sub-lethal concentrations of tetracycline have been shown to increase T3SS expression and toxicity [38] and ciprofloxacin has been demonstrated to promote swimming motility [39]. In this regard, the unexplored effects of antimicrobials might provide insight into their roles in bacterial ecology and evolution in nature [40]. For instance, antibiotic-producing microorganisms in certain communities might promote colonisation and toxicity traits of certain bacteria [38,40].

### Prevention of ExoU toxicity by tobramycin in a wound healing model

Although there was a partial observable reduction in wound expansion and cell lysis after 6 h of infection, neither moxifloxacin, ciprofloxacin or gentamycin were effective at preventing ExoU mediated cell lysis in HCE-t cells 24 h after PA103 exposure (Figure 6A). Tobramycin afforded potent protection of HCE-t towards infection and cytotoxicity, which we partially attribute to a depletion in T3SS mediated toxicity and ExoU secretion and importantly, not due to reduced bacterial expansion (Figure 6C). A previous study revealed that tobramycin was effective at reducing acute cytotoxic damage and could decrease neutrophil extracellular trap (NET) formation in a mouse keratitis model of *P. aeruginosa* infection [41]. Although the authors could not conclude the mechanism of tobramycin mitigated NET formation, our results might offer insight. Host proinflammatory signalling, induced by T3SS effectors, has been shown to potentiate deleterious effects of neutrophil infiltration leading to tissue damage [42,43]. Antimicrobials such as amoxicillin have been shown to increase NET formation [44], leading to exacerbated tissue damage, whereas gentamycin was shown to reduce NET formation [45]. This suggests that particular antimicrobials may fail in certain therapeutic circumstances, whereas other antimicrobial classes could be of benefit.

### Prevention of ExoS mediated cytotoxicity by tobramycin

Moxifloxacin, ciprofloxacin and gentamycin afforded limited protection towards ExoS-dependent cell rounding in an HCE-t cell (PA76026) infection model after 6 h (Figure 7A). After 24 h, we observed extensive wound expansion and cytotoxicity (Figure 7A,B). In contrast, tobramycin significantly ablated cell rounding, which also manifest as advanced wound healing and significantly reduced cytotoxicity after 24 h infection (Figure 7A,B). The apparent discrepancy in the action of tobramycin, when applied at sub-lethal concentrations (for *P. aeruginosa*), in regards to antimicrobial potential, is likely partially a consequence of impeded T3SS mediated cytotoxicity. However, given that the mode of action of tobramycin (and aminoglycosides in general) is to block bacterial protein synthesis by binding directly to the A-site on the 16S ribosomal RNA of the 30S ribosome, the specific mechanisms by which aminoglycosides inhibit the T3SS secretory apparatus remains to be explored. Undoubtedl,y however, interference of T3SS and thus secretion of ExoS, is likely a major contributary factor in the reduction in PA76026 cytotoxicity, at concentrations of tobramycin determined to be only minimally bactericidal in isolation. Although gentamycin is also an aminoglycoside, it was only able to reduce PcrV expression at concentrations exceeding the MIC_50_ (Figure 2B), which may explain why gentamycin offered limited protection in wound healing models. In this regard, it is noteworthy that several studies that have determined gentamycin to be less active than tobramycin [44,46].

## Conclusions

In the present study, we have demonstrated that tobramycin, although a less potent bactericidal compound *in vitro* than both moxifloxacin and ciprofloxacin, may be an effective countermeasure against *P. aeruginosa* infections through the deregulation of the T3SS pathway.

These results could indicate that, when challenged by aminoglycosides, *P. aeruginosa* is less cytotoxic, with reduced capacity for systemic spread of infection. ExoU and ExoS expressing *P. aeruginosa* from bloodstream isolates of patients with bacteraemia were distinguished to be more susceptible to aminoglycosides amikacin (100% susceptible) and gentamycin (89% susceptible) than ciprofloxacin (48% susceptible) [12]. Aminoglycosides are sometimes administered to patients with another class of antimicrobial, such as a beta-lactam, in a combinational therapeutic approach [46]. Although we did not investigate beta-lactams on TS33 or antimicrobial combinations, the results of this study would suggest that a combination of a more bactericidal antimicrobial and a T3SS inhibiting aminoglycoside such as tobramycin, might serve to improve disease treatment outcome. It also raises the intriguing possibility for more targeted therapeutics directed towards TS33 or related secretory systems. In a study of combination antibiograms, to assess the susceptibility of *P. aeruginosa* from respiratory cultures, it was revealed that beta-lactam susceptibility ranged from 58% to 69% and addition of a fluoroquinolone or aminoglycoside resulted in improved susceptibility. Importantly, however, only addition of tobramycin or amikacin provided susceptibility rates approaching or exceeding 90% [46].

## Materials and methods

### Chemicals, reagents and antibodies

Ciprofloxacin, moxifloxacin, tobramycin and gentamycin were purchased from Merck. The PcrV antibody Mab 166 was purchased from Creative Biolabs (New York, U.S.A.). The pOPINF *E. coli* expression vector was purchased from Addgene. ExoU with a C-terminal 6xHistidine tag was cloned into pUCPT20 and transformed into PA103 where indicated. LIVE/DEAD assay reagents were purchased from Invitrogen. LDH assay reagents were purchased from Thermofisher.

### Bacterial strains

The strain of *P. aeruginosa*, PA103 was gifted by Professor Dara Frank (Medical College of Wisconsin). PA76026 is a clinically genotyped and phenotyped ExoS expressing strain that was obtained from the University of Liverpool, which houses isolates of the Microbiology Ophthalmic group. The pUCPT20 encoding ExoU with a C-terminal 6xHistidine tag were transformed into PA103 by electroporation with 300 µg/ml carbenicillin employed as the selection marker.

### Recombinant protein purification

Expression of ExoU, kRas and the ADPRT domain of ExoS (residues 233–453), with N-terminal 6xHistadine tags, were induced in transformed *E. coli* (C43 (DE3) for ExoU and ExoS and BL21 Star^TM^ (DE3) for kRAS) with 0.4 mM isopropyl-β-D-thiogalactopyranoside (IPTG) when bacteria were at logarithmic growth phase (OD_600nm_ 0.6–0.8). ExoS and kRas were expressed for 16 h at 18°C and ExoU was expressed for 3 h at 30°C. Bacterial pellets were lysed by either sonication or using a Constant systems cell disruptor (at 19K Psi) in 20 mM Tris–HCl (pH 8.2), 300 mM NaCl, 0.1% (v/v) Triton-X-100, 10 mM imidazole, 1 mM DTT, 10% (v/v) glycerol and a cOmplete protease inhibitor cocktail tablet (Roche). ExoU and ExoS were purified by immobilised nickel affinity chromatography (IMAC) followed by size-exclusion chromatography (SEC) (16/600 Superdex 200, GE healthcare) in 20 mM Tris–HCl (pH 8.2), 100 mM NaCl and 10% (v/v) glycerol. After IMAC, kRas was incubated with TEV protease followed by dialysis (4°C for 16 h) then reverse purification (HisTrap column). kRas was further purified by anion exchange (HiTrap Q HP column) chromatography. Finally, a HiPrep 26/10 Desalting column was used to exchange kRas into 20 mM Tris–HCl (pH 8.0), 300 mM NaCl and 10% (v/v) glycerol buffer.

### Western blotting

Bacteria were isolated by centrifugation at 5000×***g*** for 5 min. After resuspension in lysis buffer (50 mM Tris–HCl (pH 7.4), 1% (v/v) NP-40, 0.1% (w/v) SDS, 100 mM NaCl, 1 mM DTT, 10% (v/v) glycerol and cOmplete protease inhibitor cocktail (Roche)), bacteria were briefly sonicated on ice and then centrifuged at 16 000×***g*** prior to protein quantification using the Bradford assay (Thermo Fisher). Samples were heated at 98°C for 5 min in sample buffer (50 mM Tris–HCl (pH 6.8), 1% (w/v) SDS, 10% (v/v) glycerol, 0.01% (w/v) bromophenol blue, and 10 mM DTT). Subsequently, 80 µg of total protein for each sample was resolved by SDS–PAGE prior to transfer to nitrocellulose membranes (Bio-Rad). Membranes were blocked in Tris-buffered saline with 0.1% (v/v) Tween 20 (TBS-T) in 5% (w/v) milk (pH 7.4) followed by incubation with indicated primary antibodies overnight. Proteins were detected using appropriate secondary HRP-conjugated antibodies and enhanced chemiluminesence reagent (Bio-Rad). ImageJ software [NIH (National Institutes of Health), Bethesda, MD, U.S.A.] was used to calculate the intensity of immunoreactive bands minus the background and the intensity of PcrV immunoreactivity was then divided by that of the respective RNApolβ immunoreactivity to account for any differences in sample loading.

### RT-qPCR

Bacteria were sub-cultured at OD_600nm_ ∼ 0.1 and then grown in a shaker incubator at 37°C for 16 h in the presence of indicated antimicrobial agent. Cells were collected by centrifugation and lysed in RLT buffer (Qiagen) according to the manufacturer's instructions. mRNA was extracted using an RNA extraction kit (Qiagen). Complete cDNA was generated from total RNA using GoScript Reverse Transcription system (Promega), using 1 µg RNA per reaction and 0.5 µg of Random primer. qPCR was performed in triplicate using the Comparative Ct (ΔΔCt) method on an Applied Biosystems (AB) StepOnePlus machine, a Power SYBR Green PCR Master Mix (Thermo Scientific) and the following primer pairs. Expression levels were normalised to AmpC mRNA.

*exoU*: left 5′-AGAACGGAGTCACCGAGCTA and right 5′-CGAGCAGCGAAATAAGATCC.

*exoS*: left 5′-ATGTCAGCGGGATATCGAAC and right 5′-CCTCAGGCGTACATCCTGTT.

*pcrV*: left 5′-TGATCCAGTCGCAGATCAAC and right ATCCTTGATCGACAGCTTGC.

*exsA*: left 5′-TTGAGTGAAGTCGAGCGTTG and right 5′-TCCATGAATAGCTGCAGACG.

*ampC*: left 5′-ACCCATCGCGGTTACTACAA and right 5′- GTGGAACCGGTCTTGTTCAG.

Statistical significance of differences was assessed using Student's *t*-tests for normally distributed data and performed using Prism 7 (GraphPad Software).

### *In vitro* PLA_2_ assay

ExoU sn-2 directed phospholipase activity was detected using an adapted Caymen chemical cPLA_2_ assay kit in a 96-well plate format, as previously described [29]. Assay conditions contained 1 mM Arachidonoyl thio-PC (ATPC) (Cayman Chemical, Michigan, U.S.A.), 1 µM PIP_2_ (Avanti Polar Lipids, Alabama, U.S.A.), 25 µM mono ubiquitin (Merck), 2% DMSO (v/v) and 1.25 mM 5,5-dithio-bis-(2-nitrobenzoic acid) (DTNB) (Merck) in a final volume of 50 µl. For detection of recombinant ExoU phospholipase activity, 100 nM of ExoU was added to initiate substrate hydrolysis. For detection of endogenous ExoU secreted from PA103, 10 µl of cleared culture medium, from sub-cultured PA103 in the presence of antibiotics at MIC_50_, was used. The absorbance at 405 nm (A405) was measured and background subtracted (substrate and DTNB alone) at 2 min increments over 3 h (for recombinant ExoU) and 16 h (for endogenous ExoU). Endogenous ExoU activity, after exposure of PA103 to antimicrobials, was calculated as a percentage relative to DMSO controls and normalised to the detected number of PA103 CFUs.

### *In vitro* ADP-ribosyl transferase (ADPRT) assay

Recombinant ExoS ADPRT activity was detected by monitoring conversion of 1,N^6^-etheno-NAD (ἐNAD) to 1,N^6^-etheno-ADP (ἐADP) using kRas as a substrate. Reaction condition were 100 nM ExoS, 1 µM 14-3-3ή (MRC Protein Phosphorylation and Ubiquitylation Unit), 5 µM kRas, and 25 µM εNAD^+^ (Merck) in 20 mM Tris (pH 7.4), 100 mM NaCl, 4 mM MgCl_2_ and 10 µM indicated antimicrobial. Hydrolysis of ἐNAD to ἐADP was monitored in real time using a fluorescent plate reader at 330/460 nm (Ex/Em). A calibration curve of known 1,N^6^-etheno-ADP (Merck) concentrations was used to convert fluorescence outputs in to ἐADP concentrations. For detection of native secreted ExoS enzymatic activity (from PA76026), overnight cultures were diluted (1:20) in fresh LB medium and sub-cultured with and without the indicated antibiotics (present at MIC_50_) for 16 h. Bacterial cultures were clarified by centrifugation at 5000×***g*** for 5 min after which 10 µl of the supernatant was added to 40 µl of reaction mixture (1 µM 14-3-3ή, 5 µM kRas, 25 µM ἐNAD and 4 mM MgCl_2_). After 4 h, fluorescence was detected and the percentage activity of ExoS from antibiotic treated PA76026 was calculated relative to DMSO (0.1% v/v) controls and normalised to the detected quantity of PA76026 CFUs.

### Detection of *P. aeruginosa* colony forming units

Cultures of *P. aeruginosa* in LB broth, with and without indicated antimicrobials, were centrifuged, resuspended in 1 ml of PBS, serially diluted and then incubated on agar plates overnight at 37°C prior to counting of colony forming units (CFUs). For deduction of antimicrobial MIC_50_ in a microplate format, *P. aeruginosa* growth was quantified by OD_600_ readings, which corresponded to CFU values determined from a previously established standard curve (data not shown).

### HCE-t scratch and infection assay

HCE-t cells were analysed using a scratch and infection assay as previously described [29]. Briefly, HCE-t cells were cultured to fully confluent monolayers in 24-well plates. Two parallel scratches were applied across the diameter of the wells with a pipette tip. PA103 and PA76026 were added at a multiplicity of infection (MOI) of 2.5 with the indicated antimicrobial or DMSO (0.01% v/v) controls.

### Fluorescence microscopy

Scratched and infected HCE-t cells with or without antimicrobials were incubated at 37°C in 5% CO_2_ for 24 h before analysis by fluorescent microscopy, employing Live/Dead staining (Invitrogen), to differentiate and visualise viable and dead/dying cells. Culture medium was removed from the infected HCE-t cells and washed with 1 ml of PBS three times and fresh medium containing 5 µM of both Calcein (Ex/Em 494/517 nm) and Ethidium homodimer-1(Ex/Em 528/617 nm) was applied. Images of the scratched HCE-t cells were obtained on either an Apotome Zeiss Axio Observer or a Nikon Eclipse TiE.

### LDH assays

As an indicator of cell lysis, lactate dehydrogenase (LDH) release was measured using the Pierce LDH Cytotoxicity Assay Kit (Thermo Scientific) according to the manufacturer's instructions. Fully confluent scratched HCE-t cells, cultured in 24-well plates, were infected with indicated strains of *P. aeruginosa* at an MOI of 2.5 for 24 h in the presence of indicated antimicrobial agent (0.1% (v/v) DMSO). Culture medium (50 µl) of HCE-t cells were then subject to LDH assay analysis in 96-well plates. Each assay consisted of three technical repeats and mean results were obtained from three independent experiments. The results were reported as percent LDH release normalised to a positive control (according to manufactures instructions), which gave the maximum amount of observable cell lysis in an appropriate detectable range of absorbance.

## Data Availability

The datasets generated during and analysed during the current study are available from the corresponding author on reasonable request.

## Open Access

Open access for this article was enabled by the participation of University of Liverpool in an all-inclusive *Read & Publish* agreement with Portland Press and the Biochemical Society under a transformative agreement with JISC.

## Abbreviations

ADPRT: ADP-ribosyl transferase
CFUs: colony forming units
DTNB: 5,5-dithio-bis-(2-nitrobenzoic acid)
IMAC: immobilised nickel affinity chromatography
LDH: lactate dehydrogenase
MIC: minimal inhibitory concentration
MOI: multiplicity of infection
NET: neutrophil extracellular trap
PIP_2_: phosphatidylinositol 4,5-bisphosphate
T3SS: type III secretion system
